# Functional analysis of eliciting plant response protein Epl1-Tas from *Trichoderma asperellum* ACCC30536

**DOI:** 10.1038/s41598-018-26328-1

**Published:** 2018-05-22

**Authors:** Wenjing Yu, Gulijimila Mijiti, Ying Huang, Haijuan Fan, Yucheng Wang, Zhihua Liu

**Affiliations:** 1 School of Forestry, Northeast Forestry University, 26 Hexing Road, 150040 Harbin, China; 2 Forestry Protection Institute, Heilongjiang academy of Forestry, 134 Haping Road, 150040 Harbin, China

## Abstract

Eliciting plant response protein (Epl) is a small *Trichoderma* secreted protein that acts as an elicitor to induce plant defense responses against pathogens. In the present study, the differential expression, promoter analysis, and phylogenetic tree analysis of *Epl1-Tas* (GenBank JN966996) from *T*. *asperellum* ACCC30536 were performed. The results showed *Epl1-Tas* could play an important role in the interaction between *T*. *asperellum* ACCC30536 and woody plant or woody plant pathogen. Furthermore, the effect of the *Escherichia coli* recombinant protein rEpl1-e and the *Pichia pastoris* recombinant protein rEpl1-p on *Populus davidiana* × *P*. *alba* var. *pyramidalis* (PdPap) was studied. In PdPap seedlings, rEpl1-e or rEpl1-p induction altered the expression levels of 11 genes in the salicylic acid (SA, three genes), jasmonic acid (JA, four genes) and auxin (four genes) signal transduction pathways, and five kinds of enzymes activities The induction level of rEpl1-p was significantly higher than that of rEpl1-e, indicating that rEpl1-p could be used for further induction experiment. Under 3 mg/mL rEpl1-p induction, the mean height of the PdPap seedlings increased by 57.65% and the mean lesion area on the PdPap seedlings leaves challenged with *Alternaria alternata* decreased by 91.22% compared with those of the control. Thus, elicitor Epl1-Tas could induce the woody plant resistance to pathogen.

## Introduction

A plant-mediated response has been confirmed as a component of bio-control using *Trichoderma* spp.^1–3^, for example, *T*. *virens* on cotton and maize^4^, *T*. *asperellum* on soybean^5^, and *T*. *harzianum* on beans^6^. Some small molecular weight proteins secreted by *Trichoderma* spp. can induce plant defense responses. One of the largest groups of proteins secreted by *Trichoderma* is the small secreted cysteine-rich proteins (SSCPs), which are identified as being 100 aa long and contain four or more cysteine residues^7^. SSCPs are subdivided into four groups: (i) Hydrophobins and hydrophobin-like proteins; (ii) elicitor-like proteins; (iii) proteins with similarity to MAP kinase repressed secreted protein 1 (MRSP1); and (iv) SSCPs with no attribution to any functional category^7,8^.

Eliciting plant response protein (Epl) secreted by *Trichoderma* is an elicitor-like SSCP belonging to the cerato-platanin family, and has four cysteines that form two disulfide bonds^9^. Recently, Epl was confirmed to act as an elicitor to induce plant defense responses^5^, which provided insights into the mechanisms underlying the processes of *Trichoderma*-plant recognition, defense elicitation, and induction of resistance. Small molecular protein Sm1 (also known as Epl1) from *T*. *virens* Gv29-8 is non-toxic to plants and microbes. Instead, native, purified Sm1 from *T*. *virens* Gv29-8 triggered the production of reactive oxygen species in rice and cotton seedlings, and induced the expression of defense-related genes, both locally and systemically, in cotton^10^. Overexpression of *Sm1* in *T*. *virens* Gv29-8 significantly enhanced levels of disease protection of maize seedlings challenged with the pathogen *Colletotrichum graminicola*, and this protection was associated with notable induction of jasmonic acid and green leaf volatile-biosynthetic genes, which demonstrated that the activity of a functional elicitor was required for *T*. *virens*-mediated induced systemic resistance (ISR) in maize^4^. In addition, glycosylation of *T*. *virens* Sm1 maintained the protein in a monomeric form, which elicited ISR, and deglycosylation led to the formation of an Sm1 dimer, which did not elicit ISR^11^. Similarly, overexpression of *Epl1* in *T*. *atroviride* IMI206040 promoted disease resistance against many pathogens, and induced the expression of the peroxidase and the α-dioxygenase encoding genes in tomato^12^. Moreover, the absence of *Epl-1* in *T*. *harzianum* ALL42 affected not only the recognition of *T*. *harzianum* ALL42 as a symbiotic fungus by beans^6^, but also the transcription of tomato defense-related genes during the *T*. *harzianum-*tomato interaction^13^, which indicated that Epl-1 could be important for plant protection in *Trichoderma*. Besides, *EplT4* from *T*. *asperellum* T4 was transformed into *Pichia pastoris*, and soybean leaves were protected by the recombinant EplT4 monomer against the pathogen *Cercosporidium sofinum*^5^. Epls have been studied in *T*. *virens*, *T*. *atroviride*, *T*. *harzianum*, and *T*. *asperellum*, only in the presence of herbage plant, including dicot (cotton, tomato, bean and soybean) and monocot (rice and maize) plants. However, there are no reports about the effect of Epl as an elicitor on woody plants.

In our previous study, the eliciting plant response protein gene *Epl1-Tas* from *T*. *asperellum* ACCC30536 was cloned. It encodes a protein comprising 138 aa with a molecular weight of 12.6 kDa. And the *Escherichia coli* recombinant protein rEpl1 (rEpl1-e) was obtained and could promote poplar tissue-culture seedling growth. In the present study, the transcription of *Epl1-Tas* in *T*. *asperellum* ACCC30536 was studied using quantitative real-time reverse transcription polymerase chain reaction (qRT-PCR) under eight inducing conditions. Similar sequences to *Epl1-Tas* and their promoters were analyzed in four *Trichoderma* genomes to further certify the genome wide biocontrol function of Epl1-Tas. Epl1-Tas was also recombinantly expressed in *P*. *pastoris* (rEpl1-p). Under rEpl1-e or rEpl1-p induction, the transcription of 11 genes related to hormone signaling and 5 physiological enzymes activities in *Populus davidiana* × *P*. *alba* var. *pyramidalis* (Pdpap) seedlings were detected, and the disease resistance against phytopathogen *Alternaria alternata* and the growth of PdPap transplanted seedlings were also investigated. The results could provide theoretical support and a practical reference for the development of biological small molecular protein elicitors from *T*. *asperellum* in forests.

## Results

### Promoter analysis and multiple sequence alignment of *Epl1-Tas*

The promoter of *Epl1-Tas* is 1,476 bp in length. Many cis-elements known to be involved in stress responses such as MYB, MYC, and WRKY motifs were found in the *Epl1-Tas* promoter (see Supplementary Fig. 1). The disease resistance element ‘ASF1MOTIFCAMV’ (S000024) appears three times and the disease resistance response element ‘BIHD1OS’ (S000498) appears once in promoter region of *Epl1-Tas* (Supplementary Fig. 1), suggesting that *Epl1-Tas* might be regulated by the transcription factors that bind to MYB motifs.

**Figure 1:**
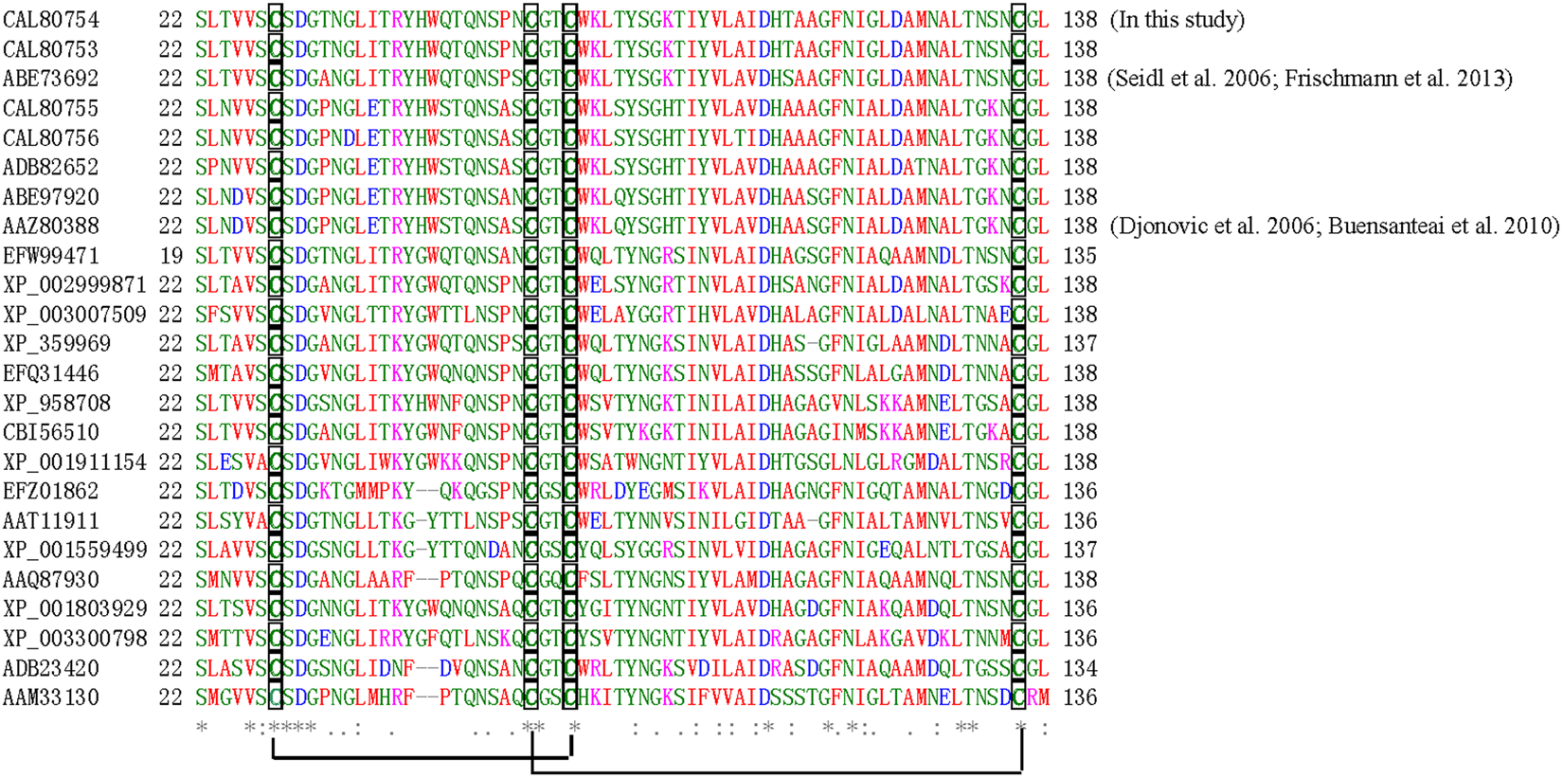
Multiple sequence alignment of sequence similar to *Epl1-Tas* from 25 fungal strains. Four highly conserved cysteine residues, C, are boxed, forming two disulfide bonds. Asterisk (*): identity. Colon (:): high similarity. Period (.): low similarity. CAL80754 and CAL80753: *T*. *asperellum*; ABE73692: *T*. *atroviride*; CAL80755 and CAL80756: *T*. *viride*; ADB82652: *T*. *reesei*; ABE97920 and AAZ80388: *T*. *viren*; XP_002999871 and XP_003007509: *Verticillium dahliae*; EFW99471: *Ophisma penicillatum*; XP_359969: *Magnaporthe oryzae*; XP_958708: *Neurospora crassa*; EFQ31446: *Glomerella graminicola*; CBI56510: *Sordaria humana*; XP_001911154: *Podospora anserina*; AAQ87930: *Cochliobolus lunatus*; XP_001559499: *Botrytis cinerea*; EFZ01862: *Metarhizium anisopliae*; XP_003300798: *Rhynchosporium secalis*; ADB23420: *Marssonina brunnea*; AAM33130: *Pectobacterium atrosepticum*; AAT11911: *Antrodia camphorate*.

Multiple sequence alignment of similar sequences from 25 fungal strains revealed that the amino acid sequences of these proteins were conserved, with identities ranging from 50 to 92%. Epl1-Tas from *T*. *asperellum* ACCC30536 shared the highest similarity (92%, E value = 7e-95) with a known elicitor Epl1 (ABF73692) from *T*. *atroviride* ATCC74058^9,14^. Furthermore, four cysteine residues that form two disulfide bonds were highly conserved among the 25 sequences (Fig. 1).

### Differential expression of *Epl1-Tas* under eight inducing conditions

**Figure 2:**
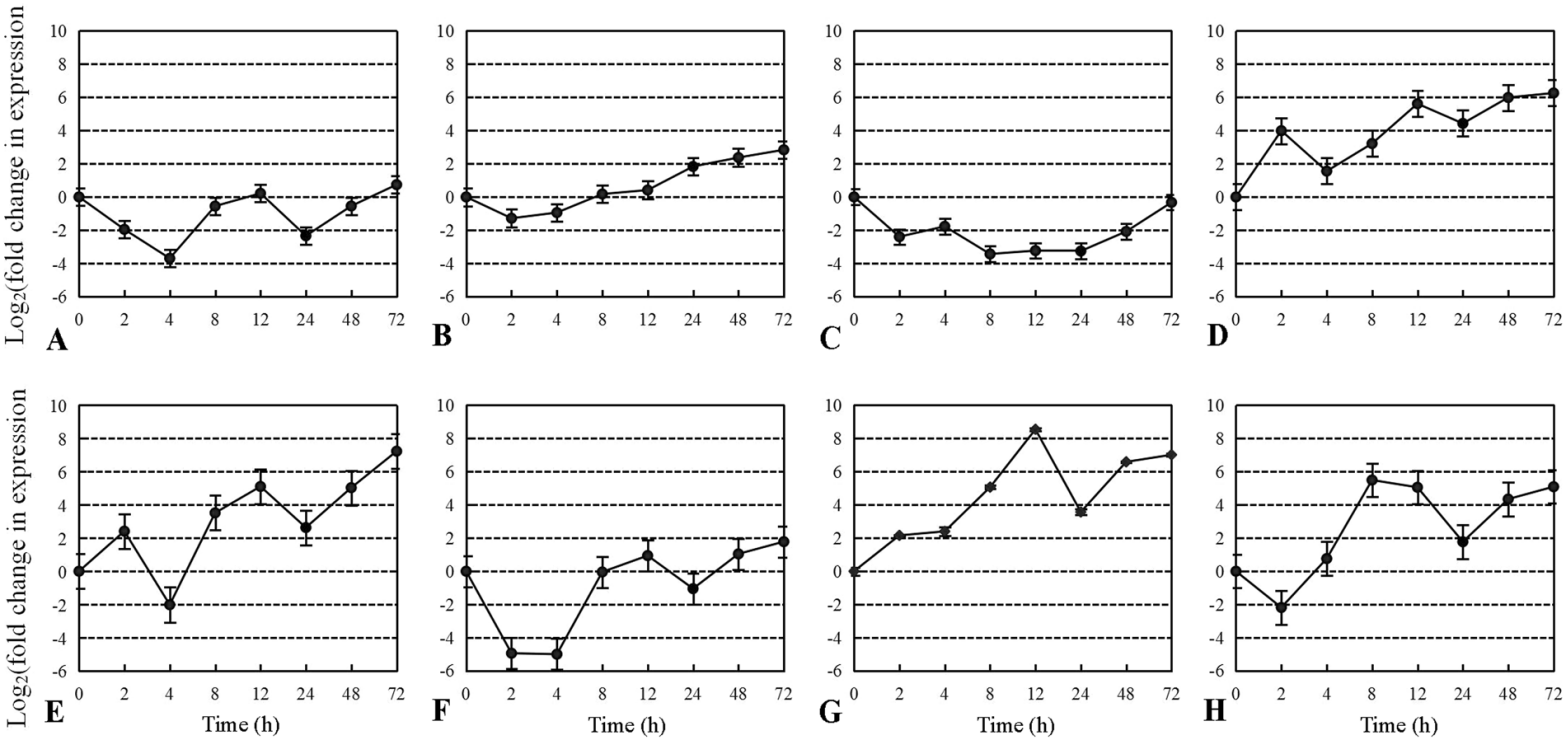
Differential expression of *Epl1-Tas* in *T*. *asperellum* ACCC30536 under eight inducing conditions. X-axis: time points, Y-axis: expression level = Log_2_(fold change in expression), namely expression of *Epl1-Tas* under minimal medium (MM) conditions (**A**), C starvation conditions (**B**), N starvation conditions (**C**), 1% mycelia powder of *A*. *alternata* grown in 1/4PD for 10 d (**D**), 5% fermentation liquid of *A*. *alternata* grown in 1/4PD for 10 d (**E**), 1% root powder of *Populus davidiana* × *P*. *alba* var. *pyramidalis* (PdPap) seedlings (**F**), 1% stem powder of PdPap seedlings (**G**), and 1% leaf powder of PdPap seedlings (**H**). All experiments were performed three times. The quantitative data are presented in Supplementary Table 1.

The response to eight biotic stresses of the eliciting plant response protein gene *Epl1-Tas* in *T*. *asperellum* ACCC30536 was investigated using qRT-PCR (Fig. 2). *Epl1-Tas* transcription was upregulated strongly at 72 h in response to both 1% *A*. *alternate* cell walls and 5% *A*. *alternate* fermentation liquid, by 76.64 (2^6.26^)-fold and 150.12 (2^7.23^)-fold, respectively (Fig. 2D,E). *Epl1-Tas* transcription was also upregulated in response to the 1% stem powder and 1% leaf powder of PdPap seedlings, by 362.04 (2^8.5^)-fold and 44.94 (2^5.49^)-fold at 12 and 8 h, respectively (Fig. 2G,H). Interestingly, the transcription of *Epl1-Tas* was mainly upregulated under carbon source starvation (Fig. 2B). However, *Epl1-Tas* transcription was inhibited by minimal medium (MM) (0.5% glucose and 0.5% ammonium sulfate), nitrogen source starvation, and 1% root powder. The lowest transcription levels were −1.7 (2^−3.70^), −1.43 (2^−3.43^) and −3.01 (2^−5.01^)-fold at 4, 8, and 4 h, respectively (Fig. 2A,C,F). These results suggested that the *Epl1-Tas* gene is closely associated with the response of *T*. *asperellum* ACCC30536 against biotic stresses.

### Sequences similar to *Epl1-Tas* and their promoters from four *Trichoderma* species genomes

**Figure 3:**
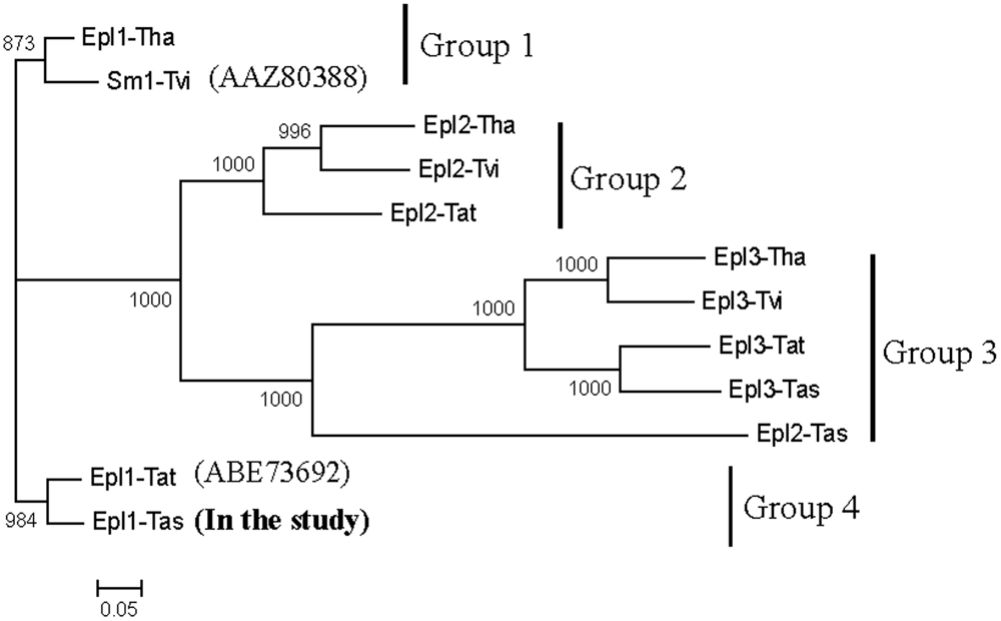
Phylogenetic tree of *Epl1-Tas* and its similar sequences. The bootstrap method was used to test the phylogeny with 1000 bootstrap replications. All branches showed significant support (>50% bootstrap values).

We screened four *Trichoderma* spp. genomes and identified twelve putative open reading frames (ORFs) (Table 1) that were predicted to be members of the small molecular cerato-platanin protein family. There were three *Epls* in each *Trichoderma* spp. genome. All the Epls were predicted to be acidic proteins, had the same number of amino acids (138 aa), and contained one intron and two exons (Table 1). They were divided into four groups by phylogenetic analysis (Fig. 3). *Epl1s* (*Epl1-Tas*, *Sm1-Tvi*, *Epl1-Tat*, and *Epl1-Tha*) from the four *Trichoderma* species were divided into two groups (Group1 and Group 4 in the phylogenetic tree), in which two *Epl1s* (*Epl1-Tat* and *Epl1-Tha*) were reported to be elicitors. The other eight *Epl2s* and *Epl3s* from the four *Trichoderma* species were placed in Group 2 and Group 3 in the phylogenetic tree (Fig. 3), which were not reported as elicitors.

**Table 1:** Similarity sequences to *Epl1-Tas* and their promoters from four different *Trichoderm*a species genomes.

Gene and Promoter	Location of gene on the chromosome	Similarity (%)	Number of exons	Number of introns	pI	MW/kDa	The cleavage site	CD
***Epl1-Tas*** Epl1-TasP	scaffold-7: 1427447–1427927 (−) 1427928–1429403 (−)	100	2	1	**6.68**	14.45	18 and 19: VSA-DT	20–138
*Epl2-Tas* Epl2-TasP	scaffold-6: 1675214–1675709 (−) 1675810–1677210 (−)	60	2	1	5.71	15.71	16 and 17: ITA-TY	19–136
*Epl3-Tas* Epl3-TasP	scaffold-7: 2033709–2034211 (+) 2032208–2033708 (+)	39	2	1	5.09	16.25	18 and 19: ASA-EI	21–137
***Sm1-Tvi*** Sm1-TviP	scaffold-3: 1718683–1719507 (−) 1719508-1721108 (−)	43	2	1	**5.76**	14.43	18 and 19: VSA-DT	20–138
*Epl2-Tvi* Epl2-TviP	scaffold-22: 139828–140472 (+) 138327-139827 (+)	57	2	1	4.84	15.19	18 and 19: ATA-TY	21–138
*Epl3-Tvi* Epl3-TviP	scaffold-3: 2346793-2347294 (+) 2345292-2346792 (+)	40	2	1	5.01	16.06	18 and 19: VSA-DI	21–137
***Epl1-Tat*** Epl1-TatP	scaffold–11: 731150-731989 (−) 731990–722490 (−)	92	2	1	**5.98**	14.55	18 and 19: VSA-DT	20–138
*Epl2- Tat* Epl2- TatP	scaffold-5: 467247–467747 (+) 465746–467246 (+)	62	2	1	5.86	15.47	18 and 19: ITA-TY	22–138
*Epl3- Tat* Epl3- TatP	scaffold-11: 1327744-1328241 (+) 1326243-1327743 (+)	38	2	1	4.38	15.89	18 and 19: ASA-ET	21–137
***Epl1-Tha*** Epl1-ThaP	scaffold-6: 778558–779312 (+) 777057–778557 (+)	86	2	1	**6.23**	14.36	No	20–138
*Epl2-Tha* Epl2-ThaP	scaffold-10: 449893–451119 (+) 448392–449892 (+)	57	2	1	4.72	15.26	17 and 18: GSA-AT	21–138
*Epl3-Tha* Epl3-ThaP	scaffold-6: 137701–138203 (−) 138204–139704 (−)	39	2	1	4.61	16.21	18 and 19: VTA-EV	24–137

Note: Epl: Eliciting plant response protein; Tas: *T*. *asperellum* ACCC30536; Tvi: *T*. *virens* Gv29–8; Tat: *T*. *atroviride* ATCC74058; Tha: *T*. *harzianum* CBS 226.95; -P: Promoter; AA: Number of amino acid; pI: Isoelectric point; MW: protein molecular weight; CD: Conserved domain; (+): Plus strand coding; (−): Minus strand coding.

**Figure 4:**
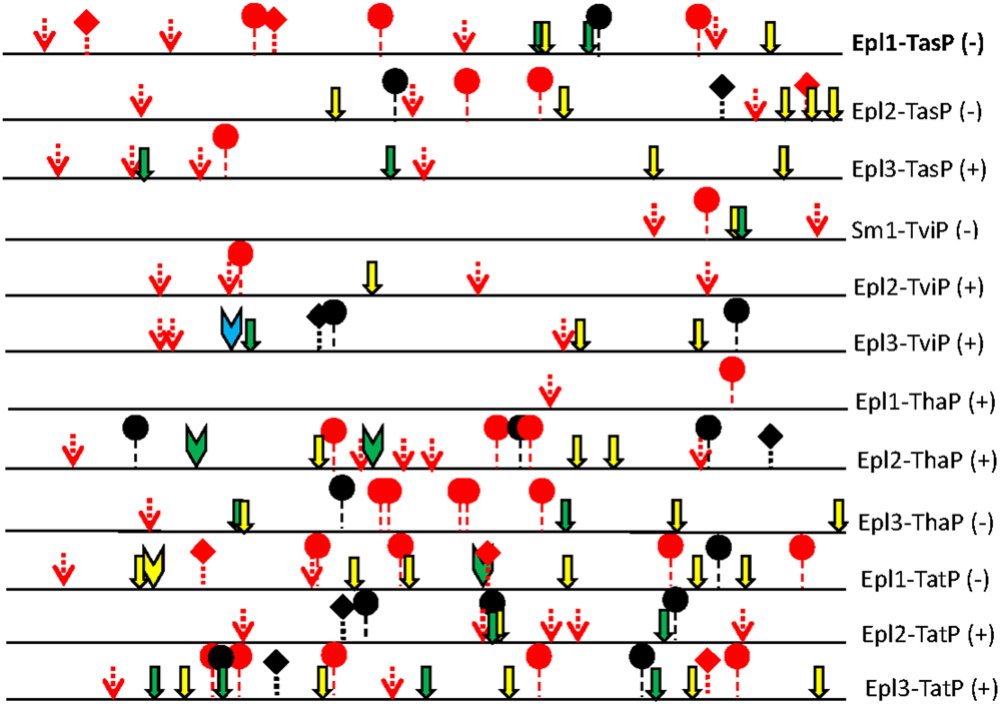
Motif analysis of promoters of *Epl1-Tas* and its similarity sequences. : putative transcription start site; : WRKY transcript factor binding site, disease resistance (ASF1MOTIFCAMV, S000024); : disease resistance responses (BIHD1OS, S000498); : low temperature responsive element (LTRECOREATCOR15, S000153); : disease mechanism, (WBOXATNPR1, S000390); : MYB transcript factor binding sit, dehydration protein (MYB2CONSENSUSAT, S000409); : Gibberellin (CCAATBOX 1, S000030); : Ethylene responsive element (ERELEE4, S000037); : Salicylic acid responsive element (GAREAT, S000142); : drought response and low temperature responsive element (DRECRTCOREAT, S000418).

Promoter analysis showed that the promoter region of *Epl1-Tas* and its similar sequences contained many stress response elements; for example, disease resistance (S000024), disease resistance responses (S000498), and drought and low temperature responsive (S000418, S000135) (Fig. 4). Moreover, many hormone response elements, such as gibberellin (S000030), ethylene (S000037), and salicylic acid (S000142), were also identified in the promoters (Fig. 4). In particular, dehydration protein (S000409) with 24.22% and disease resistance (S000024) with 19.53% appeared most frequently in the promoters, and thus might play the most important roles in the bio-control function of Epl protein. The promoter of *Epl1-Tas* contains 14 elements and thus could have more functions than the promoter of *Sm1-Tvi* (reported), which only contains five elements (Fig. 4). Moreover, the disease resistance element ‘ASF1MOTIFCAMV’ (S000024) appeared three times and the disease resistance response element ‘BIHD1OS’ (S000498) appeared once in promoter region of *Epl1-Tas*, which indicated that *Epl1-Tas* could participate in the disease resistance response.

Among the three homologous *Epl*s (*Epl1-Tas*, *Epl2-Tas* and *Epl3-Tas*) from *T*. *asperellum* ACCC30536 genome, *Epl1-Tas* has the most elements in its promoter (Fig. 4). Furthermore, only *Epl1-Tas* showed a high expression level in four inducing conditions, while *Epl2-Tas* had a lower expression level and the expression of *Epl3-Tas* barely detectable under the four inducing conditions (see Supplementary Table 2). Taken together, these results suggested that *Epl1-Tas* has multiple functions in *T*. *asperellum* ACCC30536.

### SDS-PAGE analysis of the recombinant Epl1-Tas expressed in *P*. *pastoris* (rEpl1-p)

**Figure 5:**
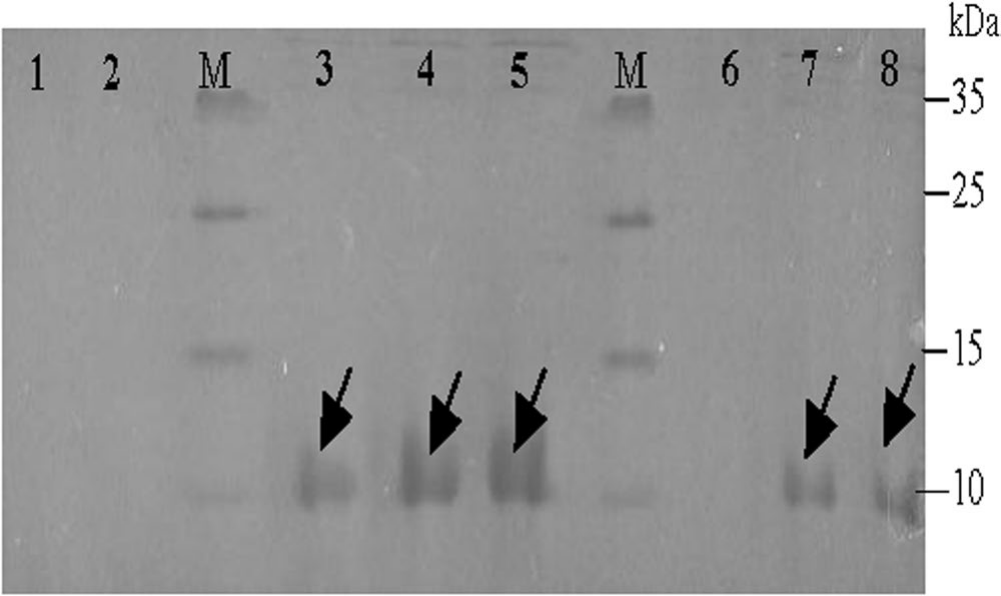
SDS-PAGE analysis of the recombinant protein rEpl1-p from GS115-Epl1. Lanes 1,2,6. The supernatant of control transformant GS115-pPIC9K induced for 6 h; lanes 3,4,5. The supernatant of transformant GS115-Epl1 induced for 2, 4, 6 h; lane 7,8. The supernatant of transformant GS115-Epl1 induced for 1 h; the concentration of methanol was 0.5% (v/v). M. Protein Marker. Full-length gels are presented in Supplementary Figure 2.

Compared with the control transformant GS115-pPIC9K, the transformant GS115-Epl1 showed a clear protein band with a molecular mass of approximately 12.6 kDa (Fig. 5). This result indicated that the recombinant protein rEpl1-p was successfully synthesized in *P*. *pastoris*.

### Effect of recombinant rEpl1-e or rEpl1-p on 11 hormone signal related genes of PdPap seedlings

**Figure 6:**
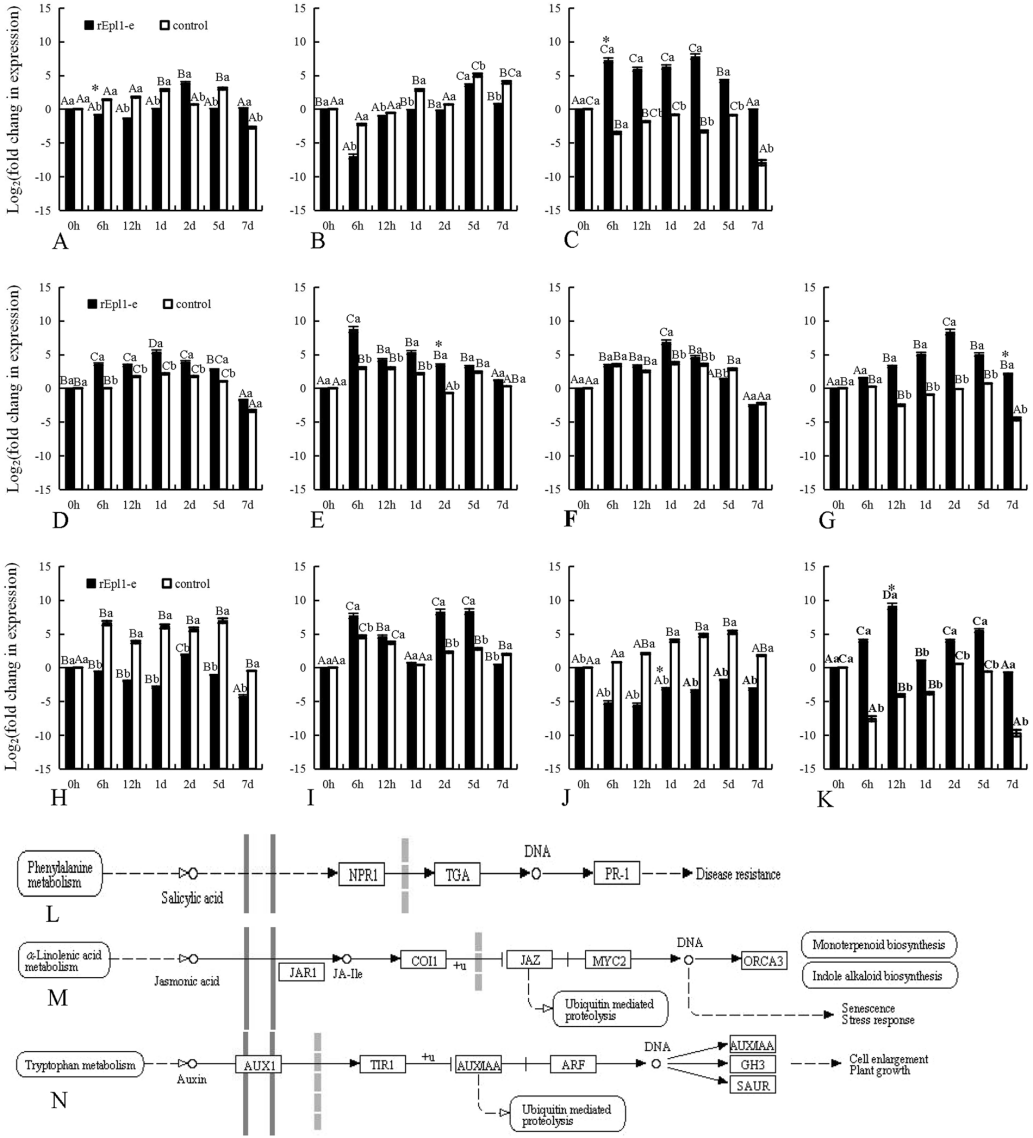
Transcription of 11 genes related to hormone signaling in *Populus davidiana* × *P*. *alba* var. *pyramidalis* (PdPap) seedlings under rEpl1-e induction. X-axis: time points, Y-axis: expression level = Log_2_ (fold change in expression). (**A**–**C**) The expression levels of *NPR1*, *TGA*, and *PR1* in the SA signal transduction pathway (**L**); (**D**–**G**). The expression levels of *COI*, *JAZ6*, *MYC2*, and *ORCA3* in the JA signal transduction pathway (**M**); (**H**–**K**). The expression levels of *TIR1*, *IAA8/AUX*, *MP/ARF*, and *GH3* in the auxin signal transduction pathway (**N**). (**L**–**N**) The plant hormone signal transduction of the KEGG software from the Kanehisa laboratory (http://www.genome.jp/kegg-bin/show_pathway?map04075). Different capital letters represent significant differences among different time points of treatment or the control group; different lowercase letter represent significant differences between the treatment and the control at the same time point; *significant difference between rEpl1-e and rEpl1-p treatments at the same time point. All significances were at *p* < 0.5. All experiments were performed three times. The quantitative data are presented in Supplementary Table 3.

**Figure 7:**
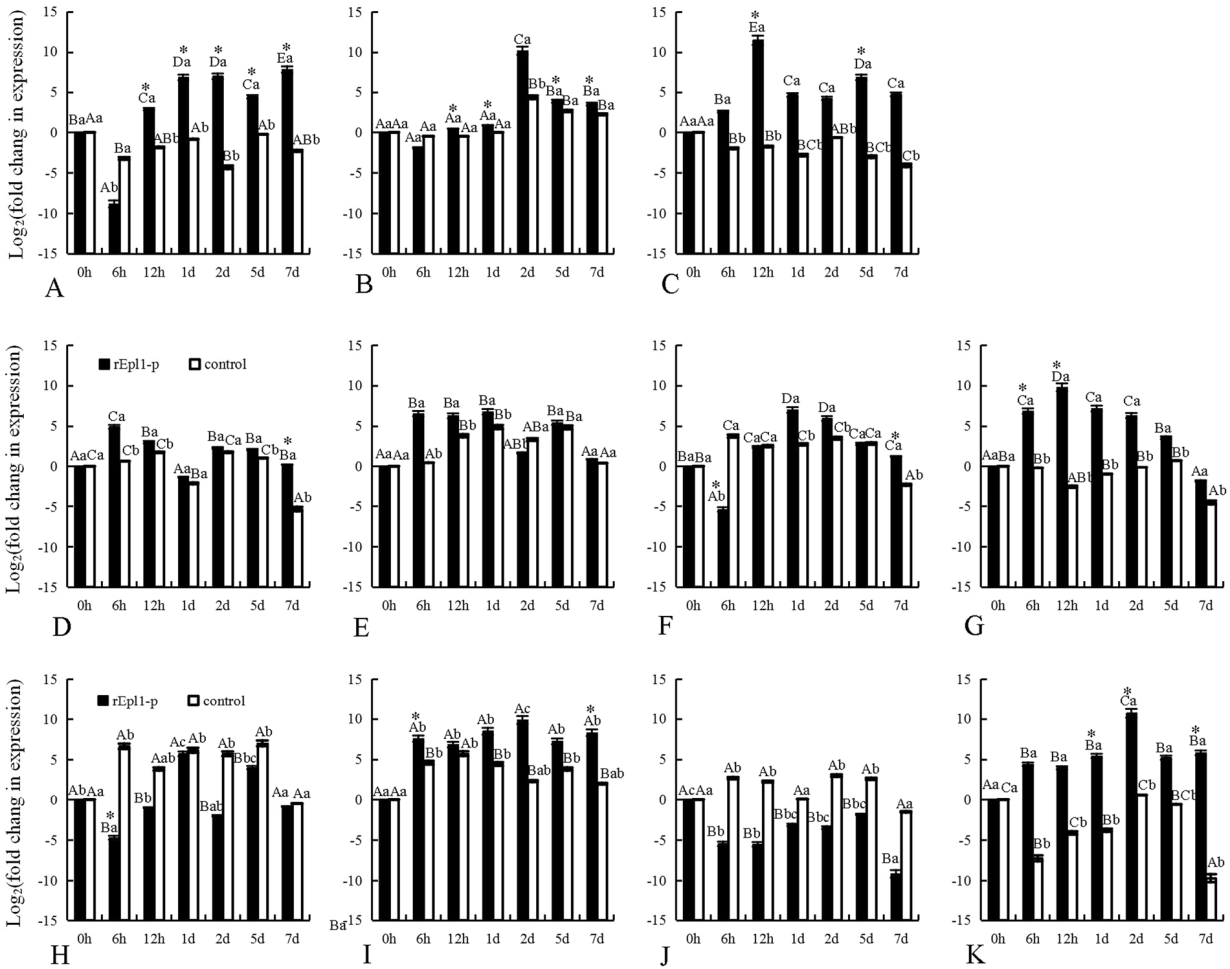
Transcription of 11 genes related to hormone signaling in *Populus davidiana* × *P*. *alba* var. *pyramidalis* (PdPap) seedlings under rEpl1-p induction. X-axis: time points, Y-axis: expression level = Log_2_ (fold change in expression). (**A**–**C**) The expression levels of *NPR1*, *TGA*, and *PR1* in the SA signal transduction pathway; (**D–G**) The expression levels of *COI*, *JAZ6*, *MYC2*, and *ORCA3* in the JA signal transduction pathway; (**H**–**K**) The expression levels of *TIR1*, *IAA8*, *MP/ARF*, and *GH3* in the auxin signal transduction pathway. Different capital letters represent significant differences among different time points of treatment or the control group; different lowercase letter represent significant differences between the treatment and the control at the same time point; *significant difference between rEpl1-e and rEpl1-p treatments at the same time point. All significances were at *p* < 0.5. All experiments were performed three times. The quantitative data are presented in Supplementary Table 4.

To determine whether the recombinant rEpl1-e (Epl1-Tas expressed by *E*. *coli* obtained in our previous study) or rEpl1-p could stimulate a response in PdPap seedlings, the transcription levels of 11 genes related to plant hormone signaling were studied using qRT-PCR (Figs 6 and 7). The transcription levels of three genes in the SA signal transduction pathway (Fig. 6L) in PdPap seedlings were significantly induced by rEpl1-e (Fig. 6A–C) and rEpl1-p (Fig. 7A–C). The expression of *NPR1* (encoding nonexpresser of PR genes 1) was downregulated from 6–12 h under rEpl1-e induction, and then upregulated to the same level as the control (Fig. 6A). The expression of *NPR1* showed a transient decrease at 6 h under rEpl1-p induction, and then also increased to the same level as the control (Fig. 7A). The expression of *TGA* (encoding TGACGTCA cis-element-binding protein) was mainly downregulated under rEpl1-e and was lower than that of the control (Fig. 6B); however, the expression of *TGA* was mainly upregulated under rEpl1-e with a peak at 57.68 (2^5.85^)-fold compared with the control (Fig. 7B). Compared with the downregulated control, the expression of *PR1* (encoding pathogenesis-related 1) was upregulated under rEpl1-e (Fig. 6C) and rEpl1-p (Fig. 7C), by 2210.26 (2^11.11^) and 9607.86 (2^13.23^)-fold compared with the control at 2 d and 12 h, respectively.

The transcription levels of four genes in JA signal transduction pathway (Fig. 6M) in PdPap seedlings were significantly induced by rEpl1-e (Fig. 6D–G) or rEpl1-p (Fig. 7D–G). The expression levels of *COI*, *JAZ6* and *MYC2* (encoding cytochrome oxidase I, jasmonate-zim-domain protein 6, and MYC-related transcriptional activator, respectively) were mainly upregulated under rEpl1-e (Fig. 6D–G) or rEpl1-p (Fig. 7D–F) induction. The peaks were 11.88 (2^3.57^), 53.08 (2^5.73^) and 8.63 (2^3.11^)-fold higher, respectively, than those of the control at 6 h, 6 h, and 1 d under rEpl1-e induction (Fig. 6D–F), and the peaks respectively were 18.90 (2^4.24^), 67.65 (2^6.08^) and 5.43 (2^2.44^)-fold higher, respectively, than the control at 6 h, 6 h, and 1 d under rEpl1-p induction (Fig. 7D–F). Compared with the downregulated control, the expression level of *ORCA3* (encoding octadecanoid-derivative responsive catharanthus AP2-domain 3) was upregulated under both rEpl1-e (Fig. 6G) and rEpl1-p (Fig. 7G) induction, and the peaks were 349.71 (2^8.45^) and 5007.93 (2^12.29^)-fold higher than those of the control at 2 d and 12 h, respectively.

The transcription levels of four genes in the auxin signal transduction pathway (Fig. 6N) in PdPap seedlings were also significantly induced by rEpl1-e (Fig. 6H–K) and rEpl1-p (Fig. 7H–K). The expression of *IAA*8*/AUX* (encoding auxin/indole-3-acetic acid 8) was mainly upregulated under both rEpl1-e (Fig. 6J) and rEpl1-p (Fig. 7I) induction, and the peaks were 63.56 (2^5.99^) and 188.71 (2^7.56^)-fold higher than those of the control at 2 d and 2 d, respectively. Compared with the upregulated control, the expression levels of *TIR1* (encoding transport inhibitor response 1 and *MP/ARF* (encoding monopteros/auxin respnse factor) were downregulated under both rEpl1-e (Fig. 6H,J) and rEpl1-p (Fig. 7H,J) induction. Compared with the downregulated control, the expression level of *GH3* (encoding Gretchen Hagen 3) was upregulated under both rEpl1-e (Fig. 6K) and rEpl1-p (Fig. 7K) induction and the peaks were 3040.30 (2^11.57^) (Fig. 6K) and 46663.28 (2^15.51^) (Fig. 7K)-fold higher than those of the control at 6 h, respectively. In summary, the recombinant rEpl1-e and rEpl1-p had an important influence on PdPap seedlings’ hormone signaling. The inducing effect of rEpl1-p was significantly higher than that of rEpl1-e, based on significance analysis.

### Effect of recombinant rEpl1-e or rEpl1-p on enzymes activities in PdPap seedlings

**Figure 8:**
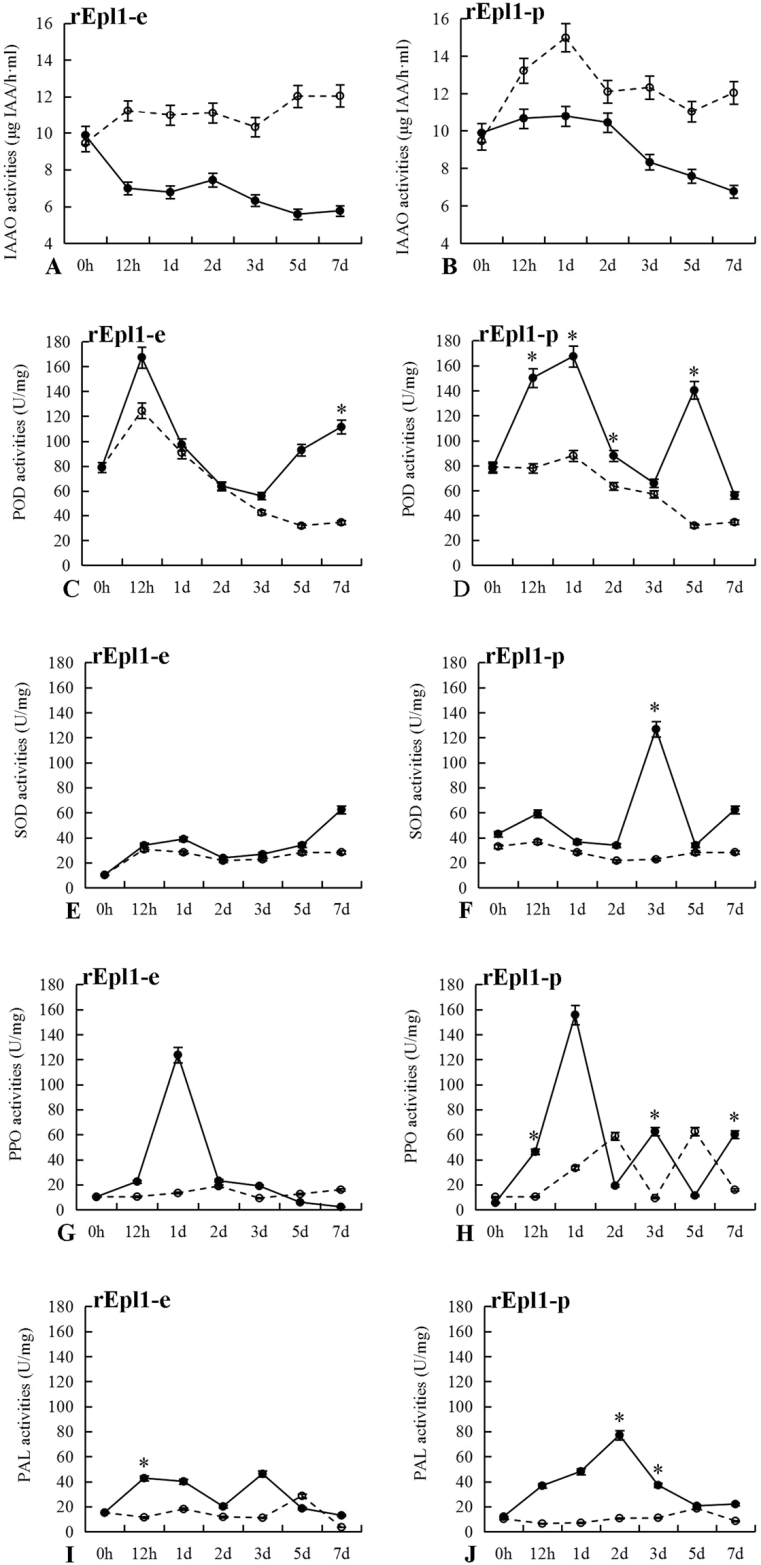
The activities of five kinds of enzymes in *Populus davidiana* × *P*. *alba* var. *pyramidalis* (PdPap) seedlings leaves under rEpl1-e or rEpl1-p induction. X-axis: time points, Y-axis: enzyme activities, namely IAAO (**A**,**B**), POD (**C**,**D**), SOD (**E**,**F**), PPO (**G**,**H**), and PAL (**I**,**J**) activities in PdPap seedlings leaves under rEpl1-e or rEpl1-p induction, respectively. Solid lines: the treatment group; dotted line: the control group. *Significant difference between rEpl1-e and rEpl1-p treatments at the same time point (*p* < 0.5). All experiments were performed three times.

To gain a further insight into the PdPap seedlings response to rEpl1-e or rEpl1-p, the activities of five kinds of enzymes in the PdPap seedlings were analyzed under rEpl1-e or rEpl1-p induction (Fig. 8). The enzyme activities showed obvious differences under rEpl1-e or rEpl1-p induction. Compared with the control, the activities of the growth-related indole acetic acid oxidase (IAAO) in PdPap seedlings slowly decreased under rEpl1-e (Fig. 8A) or rEpl1-p (Fig. 8B) induction. In addition, the activities of the defense-related peroxidase (POD), superoxide dismutase (SOD), polyphenol oxidase (PPO) and phenylalanine ammonia lyase (PAL) in PdPap seedlings were all higher than those of the control under rEpl1-e or rEpl1-p induction (Fig. 8C–J). The peaks under rEpl1-e induction were 77.01, 34.08, 110.42, and 66.37-fold higher than the control at 7, 7, 1 and 3 d, respectively (Fig. 8C,E,G,I). The peaks under rEpl1-p induction were 108.53, 104.06, 122.42, and 41.43-fold higher than the control at 5, 3, 1, and 7 d, respectively (Fig. 8D,F,H,J). In summary, recombinant rEpl1-e and rEpl1-p had important effects on growth-related and defense-related enzymes activities in PdPap seedlings. The induction effect of rEpl1-p was also significantly higher than that of rEpl1-e, based on significance analysis.

### Effect of rEpl1-p on growth and disease resistance of PdPap transplanted seedlings

**Figure 9:**
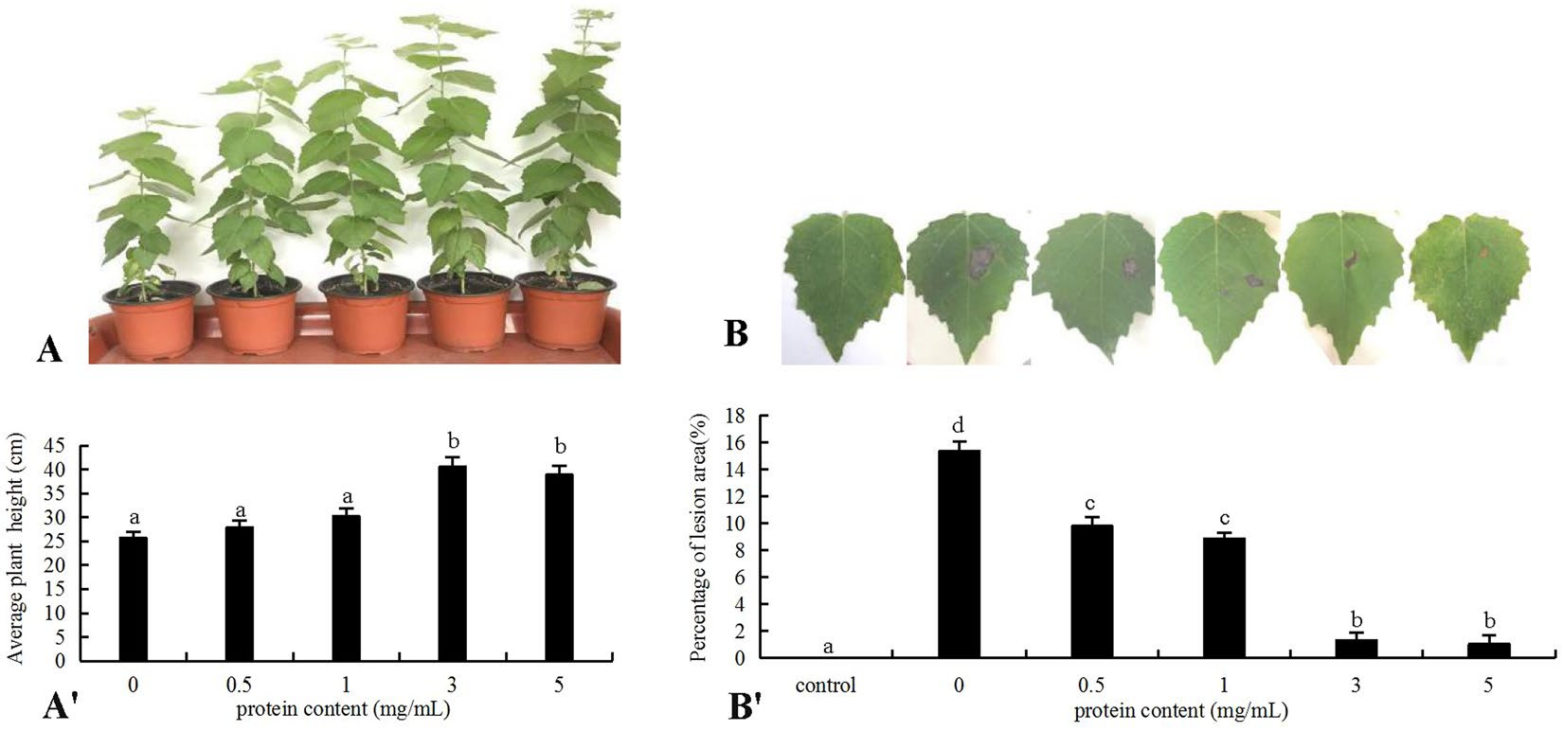
Growth and disease resistance of the *Populus davidiana* × *P*. *alba* var. *pyramidalis* (PdPap) transplanted seedlings under recombinant rEpl1-p induction. (**A**,A**′**): Differential growth rate and mean plant height of PdPap transplanted seedlings under different concentrations rEpl1-p induction; (**B**,B′): Disease-resistance ability and mean lesion area on leaves of PdPap transplanted seedlings challenged with *A*. *alternata* under different concentrations rEpl1-p induction. Different letters represent significant differences under different protein content inductions (P < 0.05). Each treatment contained five repetitions, and the experiment was repeated twice.

To determine the ability of Epl1-Tas to promote the growth and induce disease resistance of PdPap, different concentrations rEpl1-p were used to induce PdPap transplanted seedlings (Fig. 9). The growth rate of the PdPap transplanted seedlings varied under the different concentrations of rEpl1-p (Fig. 9A). Compared with the control, the mean heights of the PdPap transplanted seedlings increased significantly under induction by 3 and 5 mg/mL rEpl1-p, by 57.65% and 51.18%, respectively (Fig. 9A’).

Moreover, the disease-resistance ability of the PdPap seedlings against A. alternate also varied under the different concentrations rEpl1-p (Fig. 9B). Compared with the control, the mean lesion areas of leaves decreased significantly under 0.5, 1, 3, or 5 mg/mL rEpl1-p induction (Fig. 9A’). Especially, under induction by 3 and 5 mg/mL rEpl1-p, the mean lesion areas of leaves decreased by 91.22% and 93.17%, respectively (Fig. 9B’). Thus, rEpl1-p promoted PdPap transplanted seedlings growth and induced resistance to the pathogen *A*. *alternata* in PdPap seedlings, especially at 3 mg/mL.

## Discussion

Previously, differential expression of *Epl1* in *Trichoderma* was studied only in the presence of dicot and monocot herbage plants^4,9,10^. *Sm1* (*Epl1*) from *T*. *virens* Gv29-8 was upregulated in the presence of the cotton and maize plants early in the interaction^4,10^, and *Sm1* from different *Trichoderma* showed increased expression in presence of bean seeds^15^. In the present study, we also showed that *Epl1-Tas* from *T*. *asperellum* ACCC30536 was upregulated in the presence of the woody plant PdPap seedlings, stems, and leaves (Fig. 2G,H), which, combined with the results of previous studies, suggested that upregulated expression of *Epl1* participates in the interaction between *Trichoderma* and plants ubiquitously. However, this increase in the expression of *Epl1-Tas* was not observed in the presence of plant roots, as it was for *Sm1* from *T*. *harzianum* T37^15^ (Fig. 2F), Furthermore, the transcription of *Epl1* from *T*. *atroviridis* ATCC74058 was induced cell walls from the plant pathogen *Rhizoctonia solani*^9^. Similarly, in this study, a high transcription level of *Epl1-Tas* was induced by cell walls and fermentation liquid from the PdPap pathogen *A*. *alternata*. Thus, *Epl1-Tas* of *T*. *asperellum* ACCC30536 might not only play an important role during the interaction with herbage plants or their pathogenic fungi, but also in the interaction with woody plants and their pathogenic fungi, indicating the enriched functions of Epls.

*T*. *jecorina*, *T*. *virens*, and *T*. *atroviride* each have three paralogs of *Epls* (*sm1*)^7,14,16^. In our analysis, *T*. *asperellum* and *T*. *harzianum* also have three *Epls* (Table 1), which indicated that Epls as elicitor are ubiquitous in the *Trichoderma* genus and could be important for the bio-control of *Trichoderma*. In addition, the elicitor Sm1 from *T*. *virens* Gv29-8 (*Sm1-Tvi*) could induce local and systemic defenses in plants, including rice, cotton and maize^4,10–12,15,17^. Our analysis showed that compared with the promoter of *Sm1-Tvi*, the *Epl1-Tas* promoter has more types and numbers of regulatory motifs (6 types; 14 numbers *vs*. 4 types; 5 numbers), and the disease resistance element ‘ASF1MOTIFCAMV’ (S000024) appears three times and the disease resistance response element ‘BIHD1OS’ (S000498) appears once in promoter region of *Epl1-Tas* (Fig. 4). This indicated that *Epl1-Tas* might have more functions than *Sm1-Tvi*, and might play an important role in bio-control of *T*. *asperellum* ACCC30536, as suggested by Pozo *et al*.^18^. Furthermore, compared with its homologs in *T*. *asperellum* ACCC30536 (*Epl2-Tas* and *Epl3-Tas*), the *Epl1-Tas* promoter has more types and numbers of regulatory motifs (6 type; 14 numbers *vs*. 6 type, 13 numbers (*Epl2-Tas*) *vs*. 4 types; 9 numbers (*Epl3-Tas*)) (Fig. 4). This might explain why the expression of *Epl1-Tas* was higher than that of *Epl2-Tas* and *Epl3-Tas* under four inducing conditions (see Supplementary Table 2), which agreed with and enriched the conclusion of Frischmann *et al*.^14^.

In the phylogenetic tree, *Epl1s* (*Epl1-Tas*, *Sm1-Tvi*, *Epl1-Tat* and *Epl1-Tha*) from four species of *Trichoderma* were divided into two groups (Group1 and Group 4), in which two *Epl1s* (*Epl1-Tat* and *Epl1-Tha*) were reported to be elicitors (Fig. 3). However, the other eight *Epl2s* and *Epl3s* from the four species were placed in Group 2 and Group 3 (Fig. 3), which were not reported as elicitors. The highest expression of *Epl1-Tas* among Group 4 *Epl1s* under four inducing conditions (see Supplementary Table 2) further indicated that Epl1 proteins play an important role in different *Trichoderma*, while Epl2 and Epl3 had no obvious effect. Thus, the promoter (Fig. 4), differential expression (see Supplementary Table 2), and phylogenetic tree (Fig. 3) analyses further suggested that *Epl1-Tas* functions in stimulating plant defense responses as an elicitor.

Furthermore, to study the function of Epl1-Tas from *T*. *asperellum* ACCC30536 to the woody plant PdPap seedlings, we examined the transcription of 11 genes related to hormone signal in PdPap seedlings under *E*. *coli* recombinant rEpl1-e or yeast recombinant rEpl1-p (Fig. 5) induction, respectively. *Trichoderma* spp. colonization could induce a transient increase in expression of defense-related genes of plant^19^. That observation was in agreement with our results that 11 hormone signaling-related genes in PdPap seedlings showed transiently upregulated expression under rEpl1-e or rEpl1-p induction (Figs 6 and 7). In the SA signal transduction pathway, *NPR1* plays an important role in both innate immunity and systemic acquired resistance (SAR), and *PR1* is often associated with plant host resistance. Although *NPR1* is a key component in the induction of SAR-related *PR1* expression, its overexpression did not lead to *PR1* expression in *Arabidopsis*^20^. Similarly, although the transcription of *NPR1* in PdPap seedlings was downregulated under rEpl1-e or rEpl1-p induction (Figs 6A and 7A), *PR1* was upregulated compared with the control group, which was consistent with the results of Mou *et al*.^20^. The transient peak of *PR1* was 2210.26 (2^11.11^)-fold higher at 2 d under rEpl1-e induction (Fig. 6C), and the transient peak of *PR1* was 9607.86 (2^13.23^)-fold at 12 h under rEpl1-p induction (Fig. 7C), which showed rEpl1-e and rEpl1-p could trigger SAR in PdPap seedlings. In addition, in the JA signal transduction pathway, Octadecaniod-derivative responsive catharanthus AP2-domain protein gene *ORCA3* is a jasmonic acid responsive gene and regulates jasmonate-responsive expression, which could induce ISR in the host plant^21,22^. In this study, *ORCA3* showed higher expression in PdPap under rEpl1-e or rEpl1-p induction (Figs 6G and 7G). The highest peaks of *ORCA3* were 349.71 (2^8.45^)-fold at 2 d under rEpl1-e induction (Fig. 6G) and 5007.93 (2^12.29^)-fold at 2 h under rEpl1-p induction (Fig. 7G), respectively, which showed that rEpl1-e and rEpl1-p, similar to jasmonic acid, could also trigger ISR in PdPap seedlings. These results showed that rEpl1-e and rEpl1-p affected SA and JA signal transduction pathways in PdPap seedlings; however, the transcription level under rEpl1-p induction was significantly higher than under rEpl1-e induction.

Alterations to defense-related enzyme activities in the leaves of PdPap seedlings were detected under rEpl1-e or rEpl1-p induction. When plants suffer damage, more O^·^_2_^−^ is produced, in which case, SOD dismutates O^**·**^_2_^−^, and the subsequent excessive H_2_O_2_ induces the upregulation of *POD*, which showed that POD and SOD activities would increase to avoid injury to plant cells^23–25^. In this study, the overall trend of the POD and SOD activities in leaves of PdPap seedlings were similar under rEpl1-e or rEpl1-p induction, and their activities were obviously higher than those of the control (Fig. 8C–F). POD and PPO act synergistically during enzymatic browning because PPO can promote POD activity by generating H_2_O_2_ from the oxidation of phenolic compounds^25^. In addition, expression of *PAL* has been reported to be activated by the JA Signaling pathway^4,19,26^. These enzymes play an important role in plant defense, increasing the toxicity to invading pathogens and pests^25^. In this study, the activities of defense-related enzymes PPO and PAL in PdPap seedlings increased under rEpl1-e or rEpl1-p induction compared with those in the control group (Fig. 8G,I,H,J). Epl1-Tas as elicitor triggered ISR and SAR in PdPap seedlings resulting in the physiological response of PdPap seedlings. Moreover, the defense-related enzymes activities under rEpl1-p induction were also significantly higher than those under rEpl1-e induction. Perhaps the *E*. *coli* system expresses the target protein in inclusion bodies^27^, which can cause functional decreases after denaturation and renaturation, while the *P*. *pastoris* system has been engineered to yield high amounts of extracellularly secreted proteins with very few contaminating native proteins^17^, which explain why the yeast recombinant rEpl1-p had a better induction effect on PdPap seedlings. Therefore, we chose rEpl1-p further to induce PdPap transplanted seedlings challenged with pathogen *A*. *alternata*.

In induction test, the mean heights of the PdPap transplanted seedlings increased significantly under induction by 3 and 5 mg/mL rEpl1-p, by 57.65% and 51.18%, respectively (Fig. 9A,A’). This was consistent with the changes of auxin signal related genes (Fig. 7H–K) and IAAO activity (Fig. 8B) in PdPap seedlings leaves under rEpl1-p induction. Auxin acts by directly binding to the *TIR1/AFB* proteins, and promoting the degradation of transcriptional repressors called *IAA/AUX* proteins^28^. The original auxin response gene, *GH3*, could enhance sensitivity to IAA in root inhibition assays^29^. The relationship between the levels of IAA and IAAO activity is negatively correlated^30^. Auxin can regulate auxin-responsive genes^31^. Epl1-Tas, like auxin, could promote PdPap transplanted seedling growth. Moreover, the stronger transplanted seedlings had the stronger disease resistance. Under 3 and 5 mg/mL rEpl1-p induction, the mean leaf lesion areas decreased significantly, by 91.22% and 93.17% compared with that of the control, respectively (Fig. 9B,B’), which provided further evidence that the elicitor of defense response, Epl1-Tas, could induce protection of PdPap seedlings against attack by *A*. *alternata*.

In summary, the transcription of *Epl1-Tas* could be induced by woody plant PdPap seedlings stems and leaves and by the cell wall and fermentation liquid of its pathogen *A*. *alternata*. Disease resistance element ‘ASF1MOTIFCAMV’ (S000024) and disease resistance response element ‘BIHD1OS’ (S000498) were found in the promoter region of *Epl1-Tas*. *Epl1-Tas* was also successfully expressed in *P*. *pastoris* GS115. The recombinant proteins rEpl1-e and rEpl1-p both affected the transcription of 11 genes related to JA, SA, and auxin signal transduction pathways and the activities of five enzymes in seedlings of the woody plant PdPap. However, rEpl1-p had a better induction effect than rEpl1-e, making it more suitable for woody plant stimulation. Furthermore, rEpl1-p not only promoted PdPap transplanted seedling growth, but also protected PdPap transplanted seedling against attack by the pathogen *A*. *alternata*. This study increased our understanding of the role of Epl1-Tas in biocontrol and provides a practical reference for applications of Epl1-Tas in forests.

## Materials and Methods

### Strains, vectors and plant materials

*T*. *asperellum* ACCC30536 was obtained from the Agricultural Culture Collection of China (ACCC). *P*. *pastoris* GS115 and vector pPIC9K (Invitrogen, USA) were used for eukaryotic expression. Aseptic *Populus davidiana* × *P*. *alba* var. *pyramidalis* (PdPap) tissue-culture seedlings were used for the induction experiment. The stems, roots, and leaves of aseptic PdPap seedlings were used as inducing substrates for *T*. *asperellum* ACCC30536. Poplar pathogenic fungus *A*. *alternata* (the causative agent of poplar leaf wither) was used to prepare a carbon source in the inducing media for *T*. *asperellum*.

### Promoter analysis and multiple sequence alignment of *Epl1-Tas*

The 1,500 bp upstream fragments of the coding regions were obtained as predicted promoter regions. Regulatory motifs in these regions were predicted using the Promoter Database of Saccharomyces cerevisiae (http://rulai.cshl.edu/SCPD/) and the STRING tool^32^.

Proteins similar to Epl1-Tas were obtained using BlastP at NCBI (http://blast.ncbi.nlm.nih.gov/Blast.cgi). The multiple sequence alignment was conducted using the ClustalX program (http://www.ebi.ac.uk/Tools/ clustalw2/)^32^.

### Differential expression of *Epl1-Tas* in *T*. *asperellum* under eight inducing conditions

The transcription of *Epl1-Tas* in *T*. *asperellum* ACCC30536 under eight inducing conditions was studied, namely minimal medium (MM) with 0.5% (w/v) glucose and 0.5% (w/v) ammonium sulfate^33^, carbon starvation medium, nitrogen starvation medium, and variable carbon source in MM as follows: 1% (w/v) root powder, 1% (w/v) stem powder, or 1% (w/v) leaf powder from PdPap seedlings, 1% (w/v) powdered cell walls; and 5% (v/v) fermentation supernatant of *A*. *alternata* for 10 d. Root, stem and leaf powders, fungal phytopathogen cell walls, and fungal phytopathogen fermentation supernatants were prepared according to a previous study^34^. All experiments were performed in triplicate.

Total RNA was extracted from the mycelia using the Trizol reagent (Invitrogen, Carlsbad, CA, USA) and then subjected to qRT-PCR. The qRT-PCR products were analyzed using the IQ5 real-time PCR detection system (Bio-Rad Laboratories Co., Ltd., Shanghai, China). The expression level of *Epl1-Tas* was calculated from the threshold cycle according to 2^−ΔΔCT^ method^32^. The qRT-PCR primers are shown in Supplementary Table 5.

### Sequences similar to *Epl1-Tas* and their promoters from four *Trichoderma* species genomes

The Epl1-Tas amino acid sequence was used as a query to search for similar coding sequences using tBLASTN in the genome databases of *T*. *asperellum* ACCC30536, *T*. *virens* Gv29-8, *T*. *atroviride* ATCC74058, and *T*. *harzianum* CBS 226.95 genomes (http://genome.jgi-psf.org/). Bioinformatic analysis was performed according to the methods of Fan^32^. The phylogenetic tree was constructed using the neighbor-joining method in the MEGA 5.10 program^32^. The promoter analysis method is detailed in the “Promoter analysis and multiple sequence alignment of *Epl1-Tas”* section.

### Differential expression of three *Epls* in *T*. *asperellum* ACCC30536 genome under four inducing conditions

The transcription of *Epl1-Tas* and its two homologous genes in the *T*. *asperellum* ACCC30536 genome was analyzed in C-starvation (MM with 0.5% (w/v) ammonium sulfate), N-starvation (MM with 0.5% (w/v) glucose) and SXY (variable carbon source in MM as follows: 1% (w/v) root powder, 1% (w/v) stem powder, or 1% (w/v) leaf powder from PdPap seedlings)^35^. All experiments were performed in triplicate.

### *P*. *pastoris* transformations and SDS-PAGE analysis

The ORF of *Epl1-Tas* was amplified using the primers Epl1p1: 5′-ATCGG AATTCGATACGGTCTCCTACGACAC-3′ (*EcoR*I site underlined) and Epl1p2: 5′-CGATGCGGCCGCGAGGCCGCAGTTGCTCACGGC-3′ (*Not*I site underlined), digested with the indicated enzymes, ligated into the expression vector of pPIC9K, and transferred into *E*. *coli* Top10 competent cells to select positive clones. The recombinant vector pPIC9K-Epl1 was transferred into *P*. *pastoris* GS115 to induce expression according to the instructions of the *Pichia* expression kit (Invitrogen, Cat. No. K1710-01, USA). *P*. *pastoris* transformant GS115-Epl1 and the control transformant GS115-pPIC9K (*P*. *pastoris* GS115 transformed with empty vector pPIC9K), were induced with 0.5% (v/v) methanol per 24 h at 30 °C and 200 rpm, respectively. The supernatant of the transformant cultures was harvested at 1, 2, 4 and 6 h. The supernatants were collected by centrifugation at 10,000 × g at 4 °C for 10 min. After the addition of 1× loading buffer, the supernatant or cells were boiled for 5 min, centrifuged for 10 min at 8,000 rpm and loaded into a 12% SDS-PAGE gel^36^. Recombinant *E*. *coli* proteins were purified according to a pervious study^37^.

### Differential expression of hormone signal related genes of PdPap seedlings under recombinant rEpl1-e or rEpl1-p induction

The rEpl1-e obtained in our previous study^38^ and the rEpl1-p obtained in present study were used for the induction experiment. PdPap tissue-culture seedlings were cultured in WPM medium containing 10% (v/v) recombinant rEpl1-e or rEpl1-p. The recombinant protein was heat inactivated, and the PdPap tissue-culture seedlings were cultured in WPM medium containing 10% (v/v) heat-denatured protein as the control^35^. The leaves were harvested at 0 h, 6 h, 12 h, 1 d, 2 d, 5 d, and 7 d for RNA extraction. The elongation factor (*Ef*), *β-tubulin*, and *actin* genes were used as internal references to normalize the amount of total RNA present in each reaction. The qRT-PCR primers was listed in Supplementary Table 6. All experiments were performed three times.

### Extraction and detection for enzymes of PdPap under rEpl1-e or rEpl1-p induction

The treatment of the PdPap tissue-culture seedlings with rEpl1-e or rEpl1-p is detailed in the “Differential expression of hormone signal related genes of PdPap seedlings under recombinant rEpl1-e or rEpl1-p induction” section and the leaves were harvested after induction for 0, 0.5, 1, 2, 3, 5, and 7 d. Fresh tissue (0.5 g) was weighed from the above samples in each treatment with three replicates. The sample was frozen in liquid nitrogen and ground on ice with 10 mL of 100 mM pre-cold phosphate buffer (pH 6.0) containing 1% (w/v) dithiothreitol for enzyme extraction. IAAO, POD, SOD, polyphenol oxidase^30^, and PAL were extracted and their activities determined according to previous studies^25,30^.

### *A*. *alternata* resistance assay of PdPap seedlings leaves under rEpl1-p induction

The PdPap tissue-culture seedlings were transplanted into aseptic soil for 10 d. The rhizosphere of PdPap transplant seedlings were watered using the supernatant of a GS115-Epl1 culture containing final concentrations of 0, 0.5, 1, 3 and 5 mg/mL rEpl1-p, respectively. The growth of PdPap transplanted seedlings were observed after treatment for 30 d, and the plant height of PdPap transplant seedlings was measured. Meanwhile, the same position of leaves selected randomly per plant was inoculated with *A*. *alternata* of 1 × 10^3^ spores/mL (the optimum infection spore concentration). The disease spots were observed and the lesion areas were determined using Image Pro Plus 6.0 software after 10 d^5^. Each treatment contained five repetitions, and the experiment was repeated twice.

## Electronic supplementary material

Supplementary Figure 1

Supplementary Figure 2

Supplementary Table 1

Supplementary Table 2

Supplementary Table 3

Supplementary Table 4

Supplementary Table 5

Supplementary Table 6
