# Supplementary material for: Functional analysis of eliciting plant response protein Epl1-Tas from Trichoderma asperellum ACCC30536
Source: Sci Rep. 2018 May 22;8:7974. doi: 10.1038/s41598-018-26328-1 (PMC5964103; doi:10.1038/s41598-018-26328-1)
Supplement: Supplementary file 1 — Supplementary Figure 1 [file 41598_2018_26328_MOESM1_ESM.pdf]

Functional analysis of eliciting plant response protein Epl1-Tas from *Trichoderma asperellum* ACCC30536

Wenjing Yu[1,2], Gulijimila Mijiti[1], Ying Huang[1], Haijuan Fan[1],Yucheng Wang[1], Zhihua Liu[1,*]

−1476 TTTTACTAGCAT**CAAT**GCACTGAGGTTCGTATATGC −1441

CAATBOX1

CGGCATGACTCCATCCAAGCAGTAAAAAGCCCAAGGCCT**CGGTTG**CGTAAATATATACCCAGAAGGACCTCTGTCTGCCA −1361

MYBCORE

GGAATGTGATTCTCGAAGAACAAAGGAATCGGAGAAGAGAGAGATAGGGGAAAAAAAAGAATAAGAATAAAAATTAGACG −1281

TCTCGTATTACGATCCTTTTGGGCTT**CAAT**TACATCCCACCCGTACATGCACTGCGTGCTCCGACCCATCTTTTGGCGTC −1201

CAATBOX1

TTCTAGACAAACCG**CCAACC**GTCCCATTTCTCGTACAAACTGCATCACAGGGCCGGCACACTGCGTCAT**CAAT**ACGAGCC −1121

MYBPZM **CAATBOX1**

GGATCGACACAGCGTGCGCTCCTTGCGGCTGTGGCGATGCTCCAGGGTCCTTGTCCGGGGAAAGCTGGTTGGGTGATTGG −1041

GGGGACCGTCGTCAAGAGGGAAAGAATCCCCCGATAATCCGGTAGGCCCGCTTGCAGAAGATGGTTGTTGCTAGTAGAGG −961

AACTAGCTGTAAGAATGGATCTTGAGGGCTGAAGAGGAGGTATTGTGGACTGAGAGTGAGAGGCCCAACTGAG**TGAC**GAG −881

WRKY710S
ASF1MOTIFCAMV

ATGGACTAGGACCAGAAGAG**CAAT**GGATGGACGCCTC**TGAC**AGCCATCTCTAATGC**AACGG**CAAAAATACGAGCTTAATT −801

CAATBOX1 **WRKY710S** **MYBCOREATCYC**
MYB2CONSENSUS

GCGAGTATGAATTTTAGCCCTACTTTTTTTCTTAAAAAAATCCTTTTCTTTCGGTGTAAAGCTA**TGAC**GAATTCTGAATC −721

WRKY710S
ASF1MOTIFCAMV

TCCGTATACGCCTATTCACGTATACTTCCTAACCTTATGCCAGCCATCGGACATGGACTTGTGGCCCA**GCACCACAGCAT** −641

CAAATGGCCCGTGCCGCCCATACCACGC**ACCAACC**TGATGGATCTCCCCATGGCTTCTTCCGGTACCGCGACAAACAAGT −561

Predicted transcript start
MYBPZM

TACGAGCCCCAGGTGCTCCT**TGTCA**GGCTGCTAGCTACGTCCGGGACAGGGATGCGTTGCTATTTGTGGATTTGTGGACC −481

BIHD1OS

AGAATCGGCATCAGATGATCGGACACGAGCAAAGAGCCGGATGATGCCTGAGCGCAG**TTGAC**TCGTCACAACGATTGCTA −401

WRKY710S
MYBCORE

CCCATTTACCTCCGAT**TAACTG**CATGAGGTACTAGTATCTGATATTTAGTAGGTAT**TATTCT**TCACCGGGTCATATGTAA −321

MYB2AT **-10PEHVPSB**
MYB2CONSENSUS

GTATACTTCCGAGGGAAAGAAGAGGGGCTTGGC**TGAC**TGGGATGCAGCAGGCGGGAGGACTTGGCGATGGAT**GGATA**CCA −241

WRKY710S **MYBST1**

CGATCCCATCGAACGAGAAGGAAGAGGCGACTGAGTTTCGACGGACAAGTCCCAA**AACGG**CCA**TGAC**GGGCAAG**TTGCTC** −161

WRKY710S
MYBCOREATCYC **ASF1MOTIFCAMV**

TGAGTATAAAAACCAGACTCGTCCTCGCCTGAGA**AACCA**ATTGCTTTGAACCATCATCTTCAGTCTTCATCACAACATCA −81

MYB1AT

ACCGGCCCAAGCCTCCTCACAACACATTCGCTTTAATACTCAGCTTTATCTAATATCCATACCTCGACAACACAGTCAAG −1

Supplementary Figure 1 The promoter sequence of *Epl1-Tas* gene from *T. asperellum* ACCC30536
