# Supplementary material for: Functional analysis of eliciting plant response protein Epl1-Tas from Trichoderma asperellum ACCC30536
Source: Sci Rep. 2018 May 22;8:7974. doi: 10.1038/s41598-018-26328-1 (PMC5964103; doi:10.1038/s41598-018-26328-1)
Supplement: Supplementary file 3 — Supplementary Table 1 [file 41598_2018_26328_MOESM3_ESM.pdf]

Functional analysis of eliciting plant response protein Epl1-Tas from *Trichoderma asperellum* ACCC30536

Wenjing Yu[1,2], Gulijimila Mijiti[1], Ying Huang[1], Haijuan Fan[1],Yucheng Wang[1], Zhihua Liu[1,*]

Supplementary Table 1. Expression level of *Epl1-Tas* in *T. asperellum* ACCC30536 under eight inducing conditions

Induction time (h)	MM	C starvation	N starvation	1% mycelia powder of *A. alternata*	5% fermentation liquid of *A. alternata*	1% roots powder of PdPap seedlings	1% stems powder of PdPap seedlings	1% leaves powder of PdPap seedlings
0	0.00	0.00	0.00	0.00	0.00	0.00	0.00	0.00
2	−1.95 ± 0.10	−1.28 ± 0.04	−2.39 ± 0.07	3.99 ± 0.05	2.41 ± 0.07	−4.94 ± 0.10	2.16 ± 0.04	−2.18 ± 0.04
4	−3.70 ± 0.18	−0.94 ± 0.13	−1.77 ± 0.07	1.55 ± 0.03	−2.017 ± 0.17	−5.00 ± 0.15	2.40 ± 0.26	0.76 ± 0.09
8	−0.55 ± 0.26	0.18 ± 0.08	−3.43 ± 0.09	3.22 ± 0.09	3.52 ± 0.09	−0.04 ± 0.06	5.06 ± 0.11	5.49 ± 0.11
12	0.22 ± 0.08	0.41 ± 0.07	−3.23 ± 0.02	5.61 ± 0.05	5.10 ± 0.09	0.94 ± 0.13	8.55 ± 0.09	5.05 ± 0.08
24	−2.34 ± 0.27	1.83 ± 0.18	−3.26 ± 0.21	4.43 ± 0.13	2.63 ± 0.23	−1.05 ± 0.19	3.57 ± 0.17	1.78 ± 0.13
48	−0.55 ± 0.02	2.37 ± 0.04	−2.08 ± 0.05	5.99 ± 0.08	5.03 ± 0.05	1.04 ± 0.05	6.59 ± 0.04	4.34 ± 0.05
72	0.74 ± 0.14	2.84 ± 0.15	−0.33 ± 0.19	6.26 ± 0.13	7.23 ± 0.13	1.78 ± 0.23	7.01 ± 0.10	5.09 ± 0.08

Note: Expression level=Log_2(fold change in expression); ± standard deviation; MM, minimal medium; PdPap; *Populus davidiana* × *P. alba* var. *pyramidalis.*
