# Supplementary material for: Functional analysis of eliciting plant response protein Epl1-Tas from Trichoderma asperellum ACCC30536
Source: Sci Rep. 2018 May 22;8:7974. doi: 10.1038/s41598-018-26328-1 (PMC5964103; doi:10.1038/s41598-018-26328-1)
Supplement: Supplementary file 4 — Supplementary Table 2 [file 41598_2018_26328_MOESM4_ESM.pdf]

Functional analysis of eliciting plant response protein Epl1-Tas from *Trichoderma asperellum* ACCC30536

Wenjing Yu[1,2], Gulijimila Mijiti[1], Ying Huang[1], Haijuan Fan[1],Yucheng Wang[1], Zhihua Liu[1,*]

Supplementary Table 2. The expression of *Epl1-Tas* and its homology genes from *T. asperellum* ACCC30536 under four inducing conditions

Gene name	Transcript ID	MM	C-starvation	N-starvation	SXY
Epl1-Tas	e_gw1.7.1220.1	5940	9548	207	**26677**
Epl2-Tas	e_gw1.6.1674.1	6	23	5	1165
Epl3-Tas	e_gw1.7.1462.1	0	0	0	0

MM, minimal medium; SXY, variable carbon source in MM as follows: 1% (w/v) root powder, 1% (w/v) stem powder, or 1% (w/v) leaf powder from *Populus davidiana* × *P. alba* var. *pyramidalis* seedlings
