# Supplementary material for: Functional analysis of eliciting plant response protein Epl1-Tas from Trichoderma asperellum ACCC30536
Source: Sci Rep. 2018 May 22;8:7974. doi: 10.1038/s41598-018-26328-1 (PMC5964103; doi:10.1038/s41598-018-26328-1)
Supplement: Supplementary file 5 — Supplementary Table 3 [file 41598_2018_26328_MOESM5_ESM.pdf]

Functional analysis of eliciting plant response protein Epl1-Tas from *Trichoderma asperellum* ACCC30536

Wenjing Yu[1,2], Gulijimila Mijiti[1], Ying Huang[1], Haijuan Fan[1],Yucheng Wang[1], Zhihua Liu[1,*]

Supplementary Table 3 Expression level of 11 genes related to hormone signal in PdPap seedlings under rEpl1-e

Gene	Treatment	Time						
		0h	6h	12h	1d	2d	5d	7d
NPR1	rEpl1-e	0.00^{Aa}	-0.85±0.70^{Ab}*	-1.41±1.40^{Ab}	0.06±1.40^{Ab}	3.88±1.81^{Ba}	0.06±0.48^{Ab}	0.17±0.60^{Aa}
	control	0.00^{Aa}	1.27±1.45^{Aa}	1.76±0.73^{Aa}	2.82±0.59^{Ba}	0.68±1.27^{Ab}	3.03±1.30^{Ba}	-2.76±0.65^{Ab}
TGA	rEpl1-e	0.00^{Ba}	-7.03±1.20^{Ab}	-1.02±1.99^{Ab}	-0.09±0.46^{Bb}	-0.20±0.11^{Ba}	3.54±0.26^{Ca}	0.78±1.26^{Bb}
	control	0.00^{Aa}	-2.29±0.80^{Aa}	-0.57±1.82^{Aa}	2.82±0.59^{Ba}	0.68±0.27^{Aa}	5.03±1.16^{Cb}	3.95±1.66^{BCa}
PR1	rEpl1-e	0.00^{Aa}	7.22± 0.38^{Ca}*	5.91±0.90^{Ca}	6.24±0.98^{Ca}	7.79±0.11^{Ca}	4.16±1.25^{Ba}	-0.02±0.56^{Aa}
	control	0.00^{Ca}	-3.54±0.85^{Ba}	-1.87±1.06B^{Cb}	-0.85±0.11^{Cb}	-3.32±0.26^{Ba}	-0.91±1.36^{Cb}	-7.96±0.57^{Ab}
COI	rEpl1-e	0.00^{Ba}	3.57±0.15^{Ca}	3.37±1.96^{Ca}	5.40±0.48^{Da}	3.89±0.14^{Ca}	2.72±0.36^{BCa}	-1.76±0.48^{Aa}
	control	0.00^{Ba}	0.03± 1.65^{Bb}	1.72±0.16^{Cb}	2.12±1.30^{Cb}	1.73±1.93^{Cb}	1.02±0.73^{Cb}	-3.38±0.52^{Aa}
MYC2	rEpl1-e	0.00^{Aa}	8.70±0.27^{Ca}	4.18±0.24^{Ba}	3.97±0.72^{Ba}	3.29±0.99^{Ba}*	3.53±1.04^{Ba}	1.17±0.76^{Aa}
	control	0.00^{Aa}	2.92±0.46^{Bb}	2.88±0.47^{Bb}	2.58±0.53^{Bb}	-0.79±0.74^{Ab}	2.29±0.83^{Ba}	0.26±0.07^{ABa}
JAZ6	rEpl1-e	0.00^{Aa}	3.35±0.31^{Ba}	3.28±9.22^{Ba}	6.81±0.30^{Ca}	4.60±0.10^{Ba}	1.38±0.39^{ABb}	-2.59±0.13^{Aa}
	control	0.00^{Aa}	3.42± 0.41^{Ba}	2.49± 0.29^{Ba}	3.70±0.73^{Bb}	3.46±0.11^{Bb}	2.80±0.20^{Ba}	-2.29±0.22^{Aa}
ORCA3	rEpl1-e	0.00^{Aa}	1.50±0.71^{Aa}	3.21±0.70^{Ba}	5.06±0.62^{Ba}	8.33±0.34^{Ca}	5.00±0.40^{Ba}	2.10±0.32^{Ba}*
	control	0.00^{Ba}	0.21±1.45^{Ba}	-2.53±0.56^{Ba}	-0.96±0.16^{Bb}	-0.13±0.21^{Bb}	0.69±0.57^{Bb}	-4.56±0.38^{Ab}
TIR1	rEpl1-e	0.00^{Ba}	-0.56±0.80^{Bb}	-1.98±0.57^{Bb}	-2.90±0.30^{Bb}	1.89±0.14^{Cb}	-1.10±0.40^{Bb}	-4.25±1.71^{Ab}
	control	0.00^{Aa}	6.59± 0.75^{Ba}	3.78± 0.51^{Ba}	6.10±0.81^{Ba}	5.66±0.59^{Ba}	6.96±0.25^{Ba}	-0.49±0.51^{Ba}
IAA8/AUX	rEpl1-e	0.00^{Aa}	7.66±1.92^{Ca}	4.59±1.33^{Ba}	0.71±1.40^{Aa}	8.26±0.28^{Ca}	8.30±0.71^{Ca}	0.46±1.03^{Bb}
	control	0.00^{Aa}	4.56±0.34^{Cb}	3.68±0.27^{Ca}	0.40±0.51^{Aa}	2.28±0.23^{Bb}	2.78±0.75^{Bb}	1.96±0.43^{Ba}
MP/ARF	rEpl1-e	0.00^{Ab}	-5.18±0.97^{Ab}	-5.54±1.79^{Ab}	-3.14±2.20^{Ab}	-3.48±0.41^{Ab}	-1.84±0.37^{Ab}	-3.19±0.79^{Ab}
	control	0.00^{Aa}	0.78±0.61^{Aa}	2.07±0.50^{ABa}	3.97±1.29^{Ba}	4.77±0.28^{Ba}	5.22±0.65^{Ba}	1.78±0.59^{Ba}
GH3	rEpl1-e	0.00^{Aa}	3.98±0.39^{Ca}	9.09±1.38^{Da}*	1.05±0.06^{Bb}	3.98±0.28^{Ca}	5.49±0.37^{Ca}	-0.69±0.41^{Aa}
	control	0.00^{Ca}	-7.58± 0.35^{Ab}	-4.13±0.71^{Bb}	-3.80±0.43^{Bb}	0.56±0.16^{Cb}	-0.59±0.63^{Cb}	-9.72±1.77^{Ab}

Note: Expression level=Log_2(fold change in expression); ± standard deviation. Different capital letters represent significant differences among different time points of treatment or the control group; different lowercase letter represent significant differences between the treatment and the control at the same time point; *: significant difference between rEpl1-e and rEpl1-p treatments at the same time point.
