# Supplementary material for: Functional analysis of eliciting plant response protein Epl1-Tas from Trichoderma asperellum ACCC30536
Source: Sci Rep. 2018 May 22;8:7974. doi: 10.1038/s41598-018-26328-1 (PMC5964103; doi:10.1038/s41598-018-26328-1)
Supplement: Supplementary file 6 — Supplementary Table 4 [file 41598_2018_26328_MOESM6_ESM.pdf]

Functional analysis of eliciting plant response protein Epl1-Tas from *Trichoderma asperellum* ACCC30536

Wenjing Yu[1,2], Gulijimila Mijiti[1], Ying Huang[1], Haijuan Fan[1],Yucheng Wang[1], Zhihua Liu[1,*]

Supplementary Table 4 Expression level of 11 genes related to hormone signal in PdPap seedlings under rEpl1-p

Gene	Treatment	Time						
		0h	6h	12h	1d	2d	5d	7d
NPR1	rEpl1-p	0.00^{Ba}	-8.86±0.09^{Ab}	2.88±0.68^{Ca*}	6.87±0.08^{Da*}	7.01±0.54^{Da*}	4.40 ±0.31^{Ca*}	7.85±0.31^{Ea*}
	control	0.00^{Aa}	-3.24±0.85^{Ba}	-1.87±1.06^{ABb}	-0.85±.11^{Ab}	-4.32±0.76^{Bb}	-0.24±1.58^{Ab}	-2.29±2.11^{ABb}
TGA	rEpl1-p	0.00^{Aa}	-1.94±0.56^{Aa}	0.44±0.31^{Aa*}	0.86±0.22^{Aa*}	10.18±0.44^{Ca}	3.84±0.58^{Ba*}	3.50±0.21^{Ba*}
	control	0.00^{Aa}	-0.48±0.30^{Aa}	-0.49±0.52^{Aa}	-0.01±0.54^{Aa}	4.33±0.40^{Bb}	2.64±0.75^{Ba}	2.22±0.22^{Ba}
PR1	rEpl1-p	0.00^{Aa}	2.56±0.46^{Ba}	11.47±0.28^{Ea*}	4.62± 0.47^{Ca}	4.20±0.61^{Ca}	6.87±1.06^{Da*}	4.70±1.13^{Ca}
	control	0.00^{Aa}	-1.98±0.80^{Bb}	-1.76±0.73^{Bb}	-2.82±0.59^{BCb}	-0.65±2.58^{ABb}	-3.03±1.30^{BCd}	-4.09±1.69^{Cd}
COI	rEpl1-p	0.00^{Aa}	4.87±1.48^{Ca}	2.98±1.37^{Ba}	-1.35±0.59^{Aa}	2.26±1.17^{Ba}	2.05±0.13^{Ba}	0.20± 1.47^{Ba*}
	control	0.00^{Ca}	0.63±2.65^{Cd}	1.72± 0.16^{Cd}	-2.12±1.3^{Ba}	1.73±1.93^{Ca}	1.02±0.73^{Cd}	-5.38±0.52^{Ad}
MYC2	rEpl1-p	0.00^{Aa}	6.51± 0.22^{Ba}	6.23±0.72^{Ba}	6.74±1.18^{Ba}	1.64±0.69^{ABb}	5.39±0.68^{Ba}	0.86±0.36^{Aa}
	control	0.00^{Aa}	0.44±0.27^{Ad}	3.77± 0.24^{Bd}	4.85±0.20^{Bd}	3.30±0.21^{ABa}	4.80±0.24^{Ba}	0.38±0.76^{Aa}
JAZ6	rEpl1-p	0.00^{Ba}	-5.42±1.10^{Ab*}	2.39± 0.50^{Ca}	6.96±0.37^{Da}	5.90±0.61D^{a}	2.77±0.69^{Ca}	1.21±0.71^{Ca*}
	control	0.00^{Ba}	3.73±0.41^{Ca}	2.49±0.29^{Ca}	2.70±0.73^{Db}	3.46±0.11^{Cb}	2.80±0.20^{Ca}	-2.29±0.22^{Ab}
ORCA3	rEpl1-p	0.00^{Aa}	6.82±0.61^{Ca*}	9.76±0.38^{Da*}	7.15±0.22^{Ca}	6.27±1.26^{Ca}	3.53±0.23^{Ba}	-1.82±0.35^{Aa}
	control	0.00^{Ba}	-0.20±1.45^{Bb}	-2.53±0.56^{ABb}	-0.96±0.16^{Bb}	-0.13±0.21^{Bb}	0.69±0.57^{Bb}	-4.56±0.38^{Ab}
TIR1	rEpl1-p	0.00^{Ab}	-4.72±0.80^{Ba*}	-1.00±0.57^{Bb}	5.65±0.30^{Ac}	-2.01±0.14^{Bab}	3.96±0.40^{Bbc}	-0.86±1.71^{Aa}
	control	0.00^{Aa}	6.59±0.33^{Ab}	3.78±0.68^{Aab}	6.10±0.75^{Ab}	5.66±0.48^{Ab}	6.96±0.41^{Ab}	-0.49±0.29^{Aa}
IAA8/AUX	rEpl1-p	0.00^{Aa}	7.54±0.26^{Ab*}	6.80±0.73^{Ab}	8.45±0.19^{Ab}	9.84±0.28^{Ac}	7.20±0.28^{Ab}	8.27±0.44^{Ab*}
	control	0.00^{Aa}	4.56±0.58^{bb}	5.68±0.36^{Ab}	4.40±0.30^{Bb}	2.28±0.16Bab	3.78± 0.81^{Bb}	1.96±0.29^{Bab}
MP/ARF	rEpl1-p	0.00^{Aa}	-5.49±1.11^{Bb}	-5.54±1.30^{Bb}	-3.14±0.13^{Bbc}	-3.48±1.09^{Bbc}	-1.84±0.79^{Bbc}	-9.19±0.73^{Ba}
	control	0.00^{Ac}	2.64±0.61^{Ab}	2.21±0.50^{Ab}	0.06± 1.29^{Aa}	2.96±0.28^{Ab}	2.56±2.65^{Ab}	-1.52±2.59^{Aa}
GH3	rEpl1-p	0.00^{Aa}	4.36±0.76^{Ba}	3.92± 0.41^{Ba}	5.40±0.40^{Ba*}	10.68±0.31^{Ca*}	5.18±0.11^{Ba}	5.78±0.21^{Ba*}
	control	0.00^{Ca}	-7.27±0.35^{Bb}	-4.13±0.71^{Cb}	-3.80±0.43^{Bb}	0.56±0.16^{Cb}	-0.59±0.63^{BCb}	-9.72±1.77^{Ab}

Note: Expression level=Log_2(fold change in expression); ± standard deviation. Different capital letters represent significant differences among different time points of treatment or the control group; different lowercase letter represent significant differences between the treatment and the control at the same time point; *: significant difference between rEpl1-e and rEpl1-p treatments at the same time point.
