# Supplementary material for: Functional analysis of eliciting plant response protein Epl1-Tas from Trichoderma asperellum ACCC30536
Source: Sci Rep. 2018 May 22;8:7974. doi: 10.1038/s41598-018-26328-1 (PMC5964103; doi:10.1038/s41598-018-26328-1)
Supplement: Supplementary file 7 — Supplementary Table 5 [file 41598_2018_26328_MOESM7_ESM.pdf]

Functional analysis of eliciting plant response protein Epl1-Tas from *Trichoderma asperellum* ACCC30536

Wenjing Yu[1,2], Gulijimila Mijiti[1], Ying Huang[1], Haijuan Fan[1],Yucheng Wang[1], Zhihua Liu[1,*]

Supplementary Table 5 The primers for qRT-PCR of *Epl1-Tas* of *T. asperellum* ACCC30536

Gene name	Primer name	Sequence (5'→3')	Tm (ºC)	Size of product (bp)
Epl1-Tas	Epl1-L	CGTCTCTGCTGATACCGTCTCG	58.7	233
	Epl1-R	CGTAGATGGTCTTGCCGCTGTA	58.5	
α-tublin	αtu-L	CACATGGTTGACTGGTGCCCTA	58.6	240
	αtu-R	CTCGCCCTCTTCCATACCCTCT	59.0	
β-tublin	βtu-L	CAAACCGCCCTGTGCTCCAT	59.0	245
	βtu-R	TCGGCTGAGGCATCCTGGTAT	58.9	
actin	Act-L	AGGCAACCTTCTCGCCAACG	59.0	256
	Act-R	TCGCTTCTCGACAATGCCAACT	58.9	
