# Supplementary material for: Functional analysis of eliciting plant response protein Epl1-Tas from Trichoderma asperellum ACCC30536
Source: Sci Rep. 2018 May 22;8:7974. doi: 10.1038/s41598-018-26328-1 (PMC5964103; doi:10.1038/s41598-018-26328-1)
Supplement: Supplementary file 8 — Supplementary Table 6 [file 41598_2018_26328_MOESM8_ESM.pdf]

Functional analysis of eliciting plant response protein Epl1-Tas from *Trichoderma asperellum* ACCC30536

Wenjing Yu[1,2], Gulijimila Mijiti[1], Ying Huang[1], Haijuan Fan[1],Yucheng Wang[1], Zhihua Liu[1,*]

Supplementary Table 6 The primers for qRT-PCR of 11 hormone related genes of PdPap seedlings

Classification	Gene	Name	Primer	Sequence (5'-3')	Size(bp)
SA singal-related genes	*NPR1*	Nonexpressor of pathogenesis-related gene	NPR-L	GGCCGACGATACTTCCCTAGTT	260
			NPR-R	TGCCCTCATAGTTTCCTGAGCT	
	TGA	Transcription factor gene	TGA -L	TCGAGCAGCTCCAGCAGTCT	232
			TGA -R	AGTGCCCGTAGCCTCCCATAA	
	PR1	Pathogenesis-related protein gene	PR1-L	ACACCACCGTGCAAGCCTATG	241
			PR1-R	CGAGCAGAGTTACGCCAAACCA	
JA singal-related genes	*COI*	Coronatine insensitive protein gene	COI-L	GAGGTACTTGTGGGTGCATGGT	248
			COI-R	ACGCAGAACCTACCCTCGCT	
	JAZ6	The jasmonate ZIM-domain gene	JAZ-L	AGTGGTGAACCAAGCGAGGAG	242
			JZA-R	GGATTTGCTGCACCTTGCTGTT	
	MYC2	The helix-loop-helix domain gene	MY2-L	ACGAAGCTGCAATCTGCTGAGT	253
			MY2-R	CCAGGTCTCTCAAAGCCGACAT	
	ORCA3	Octadecaniod-derivative responsive catharanthus AP2-domain protein gene	ORC3-L	AGAGGAGTGAGGCAAAGACCCT	203
			ORC3-R	TCCGGCTCTCGACGCTTAGT	
Auxin singal-related genes	*TIR1*	Transport inhibitor response gene	TIR1-L	GTTGGTACGCAAAAGGTAGAGA	237
			TIR1-R	GGCTGACCATGCCAATACTAGC	
	IAA8/AUX	Indoleacetic acids protein gene	IAA8-L	CGGAGACTGGATGCTTGTTGGT	229
			IAA8-R	ACCTGAAACCTGACTCGTGCTC	
	MP/ARF	Auxin receptor F-box protein gene	MP-L	GCACATGAACAGGCAGGGTTT	214
			MP-R	ACCTGACCATGCACGACACTT	
	GH3	Auxin original response gene	GH3-L	TGATGCCCTCCTCGTGGAATAC	254
			GH3-R	GTGCCTCAAATGTGCCAGACTC	
Reference genes	*Tublin*	Reference gene	tu-1	TACCGAGGCTGAGAGTAACAT	245
			tu-2	GGACCCACAACTCATTCACAT	
	Ef	Reference gene	ef-1	TCACACCTGCCACATTGCTGT	230
			ef-2	TCTTGATGACACCAACCGCCAC	
	Actin	Reference gene	actin-1	TTCCGTTGCCCTGAGGTCCTAT	239
			actin-2	TCAGGAGGAGCAACCACCTTGA	
